# RNA-directed epigenetic silencing of Periostin inhibits cell motility

**DOI:** 10.1098/rsos.140545

**Published:** 2015-06-09

**Authors:** Nicholas C. Lister, Matthew Clemson, Kevin V. Morris

**Affiliations:** 1 School of Biotechnology and Biomolecular Sciences, The University of New South Wales, Sydney, New South Wales, Australia; 2 Molecular and Experimental Medicine, The Scripps Research Institute, La Jolla, CA 92037, USA

**Keywords:** Periostin, metastasis, RNA, transcription, gene silencing, epigenetic

## Abstract

The over-expression of Periostin, a member of the fasciclin family of proteins, has been reported in a number of cancers and, in particular, in metastatic tumours. These include breast, ovarian, lung, colon, head and neck, pancreatic, prostate, neuroblastoma and thyroid cancers. It is thought that Periostin plays a major role in the development of metastases owing to its apparent involvement in restructuring of the extracellular matrix to create a microenvironment favouring invasion and metastases, angiogenesis, independent proliferation, avoidance of apoptosis and the ability for cells to re-enter the cell cycle. As such we reasoned that targeted suppression of Periostin at the promoter and epigenetic level could result in the stable inhibition of cell motility. We find here that promoter-directed small antisense non-coding RNAs can induce transcriptional gene silencing of Periostin that results ultimately in a loss of cellular motility. The observations presented here suggest that cell motility and possibly metastasis can be controlled by transcriptional and epigenetic regulation of Periostin, offering a potentially new and novel manner to control the spread of cancerous cells.

## 1. Introduction

Periostin is an approximately 90 kDa matricellular protein capable of altering interactions between the extracellular matrix and surrounding cells [1,2]. Its structure contains a fourfold repeating domain that shows sequence homology with fasciclin 1, a protein found in insects, which interacts with integrins at the plasma membrane to accommodate motility and cell adhesion [1,3,4]. Binding of Periostin to various integrins activates the AKT/PKB and FAK-mediated signalling pathway, which promotes angiogenesis and cell survival, and is also involved in tumourigenesis by enabling enhanced tumour cell survival and proliferative and metastatic ability [1,5–7]. There is a significant correlation between high Periostin expression and poor prognosis in cancer patients [8–12], indicating its therapeutic potential as a gene-silencing target in an effort to reduce metastatic tumour formation.

**Figure 1.**
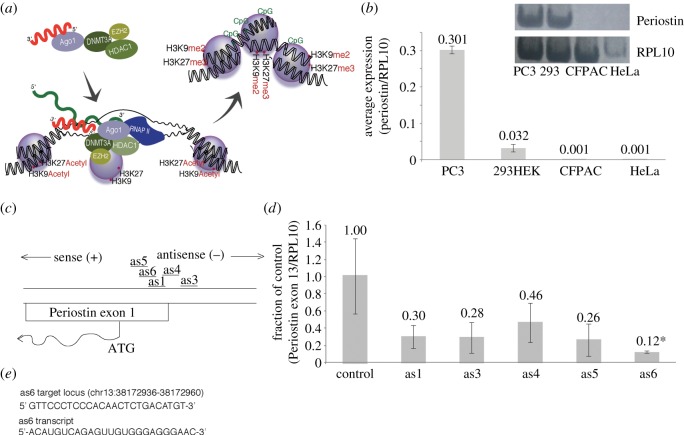
Characterization of Periostin expression and knockdown. (*a*) Schematic depicting sasRNA-directed TGS. The sasRNAs interact with Argonaute 1 (AGO1), DNA methyltransferase 3a (DNMT3a), histone deacetylase 1 (HDAC1) and Enhanzer of zeste 2 (EZH2) to epigenetically remodel target loci resulting in chromatin compaction and transcriptional silencing. (*b*) Quantitative RT-PCR (qRT-PCR) assay of endogenous expression of Periostin transcripts in various cell lines, normalized to RPL10 (*n*=3). Inset is a polymacrylamide gel of qRT-PCR products from the various cells. (*c*) Schematic depicting the sasRNA target loci in the Periostin promoter. (*d*) Periostin expression in sasRNA transfected PC3 cells 72 h post-transfection. The average of triplicate-treated cultures are shown with the standard errors of the mean and *p*-values from a paired *t*-test. Calculations are relative to parent U6M2 plasmid. (*e*) The sequences for the target locus and as6 transcript. *p<0.05 by two-tailed *t*-test. Error bars indicate s.e.m.

One pathway for inducing stable long-term transcriptional gene silencing (TGS) involves targeting a genes promoter with small antisense non-coding RNAs (sasRNAs; reviewed in [13]) (figure 1*a*). This process involves the sasRNA recruitment of epigenetic modifying complexes to the RNA targeted locus, ultimately rendering the targeted promoter loci inaccessible to RNA polymerase II (RNAPII) [14–16]. One advantage to sasRNA-directed TGS is that it can produce lasting, stable epigenetic modifications that are inherited by daughter cells [17]. As such we hypothesized that sasRNAs targeted to direct TGS of Periostin could inhibit invasive and metastatic potential of cancer cells. We report here on a sasRNA that can direct TGS of Periostin resulting in the inhibition of cell metastasis. The sasRNA reported here may prove therapeutically relevant as a means for stably silencing Periostin activity and metastasis of tumour cells.

## 2. Periostin promoter targeting with small antisense non-coding RNAs

Altered expression of Periostin is reported in a number of different forms of cancer [5,7,18]. Endogenous expression of Periostin was tested in four different cell lines. Results from qRT-PCR indicated that PC3 cells from a prostate cancer cell line had the highest expression of Periostin mRNA when compared with the other cell lines assessed (figure 1*b*). Expression was approximately 10 times greater in PC3 cells than that observed in HEK293 cells, which demonstrated the next highest expression. By comparison, CFPAC and HeLa cells showed negligible expression of Periostin. Next, we sought to determine the susceptibility of Periostin to promoter targeted sasRNAs. To determine the ability to direct TGS of Periostin, five sasRNA sequences directed to the Periostin promoter were designed (figure 1*c*), synthesized [19] and screened in PC3 cells. All five sasRNAs were capable of suppressing Periostin, with one candidate, as6, demonstrating a robust and significant repression of Periostin expression (figure 1*d*). Collectively, these data suggest that Periostin is susceptible to sasRNA-directed TGS, similar to previous observations with other genes [19–31].

## 3. Periostin knockdown is a result of small antisense non-coding RNA-directed transcriptional gene silencing

**Figure 2.**
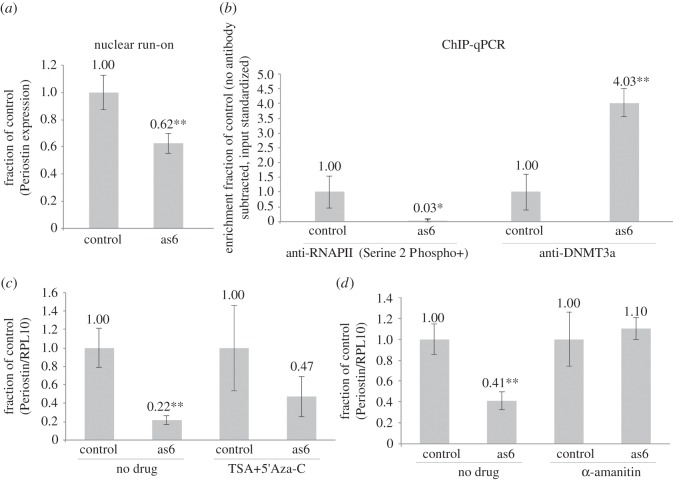
Periostin TGS in the nucleus. (*a*) Nuclear run-on analysis of Periostin transcripts 72 h after transfection with as6, normalized to RPL10 (*n*=3). Samples are Periostin transcripts 72 h after transfection with as6 (*n*=3). (*b*) ChIP of RNAPII and DNMT3a at the Periostin promoter was determined 72 h post-transfection. The relative enrichment of RNAPII and DNMT3a was determined following subtraction of no antibody beads alone control and standardized to input. (*c*) Drug treatment using both TSA and Aza-C. Drugs were added every 24 h to cultures treated as described above (*n*=3), normalized to RPL10. (*d*) Drug treatment using *α*-amanitin. Drug was added once as described above (*n*=3). All experiments were performed in PC3 cells. Throughout the figure, *p<0.05, ^**^p<0.01 by two-tailed *t*-test. Calculations are relative to parent U6M2 plasmid. Error bars indicate s.e.m.

The target sequence for as6 in the Periostin promoter overlaps the 5′UTR of Periostin (figure 1*e*) and the observed suppression of Periostin by as6 may be post-transcriptional in nature. In order to confirm that the observed reduction of Periostin mRNA is a result of TGS directed by the sasRNA as6 guide, as has been observed previously with other sasRNA-targeted loci [13,19,32], an in-depth investigation into the nuclear expression of Periostin was conducted. Nuclear run-on analysis of as6-treated cultures indicated that the observed suppression was transcriptional in nature (figure 2*a*), suggesting that as6 targeting of the Periostin 5′UTR is sufficient for modulating the transcriptional activity of Periostin in PC3 cells. SasRNA-directed TGS has been observed to result in transcriptional and epigenetic changes at the sasRNA target locus, that ultimately result in a loss of active forms of RNA polymerase II (RNAPII) and a gain of DNA methyltransferase 3a (DNMT3a) at the targeted promoter (reviewed in [13,14]). To determine if as6 treatment affects the localization of RNAPII or DNMT3a to the Periostin promoter, a chromatin-immunoprecipitation (ChIP) was performed in as6-treated PC3 cells. ChIP pull-down for active forms of RNAPII revealed a significant reduction in RNAPII and approximately fourfold increased enrichment for DNMT3a at the Periostin promoter (figure 2*b*).

Previous studies have observed that TGS can be inhibited by trichostatin A (TSA) and 5′-azacytidine (5′Aza-C), which inhibit the TGS-associated proteins histone deacetylase (HDAC) and DNMT3a, respectively [29]. Treatment of as6-transfected PC3 cells with TSA and 5′Aza-C partially restored Periostin gene expression (figure 2*c*). SasRNA-directed TGS has been observed to also require transcription at the targeted promoter and a promoter-associated RNA [16,33]. When cells were treated with the drug *α*-amanitin [32,34], an inhibitor of RNAPII, a complete reversion of mRNA expression is observed (figure 2*d*). These data suggest that TGS of Periostin requires RNAPII mediated transcription of the sasRNA guide. Collectively, the observations reported here indicate that as6 is functioning to direct TGS to the Periostin promoter in a manner similar to what has been observed previously with other sasRNA-targeted genes [20,21,32,35] (reviewed in [13,14]).

### 3.1 as6-mediated phenotypes

**Figure 3.**
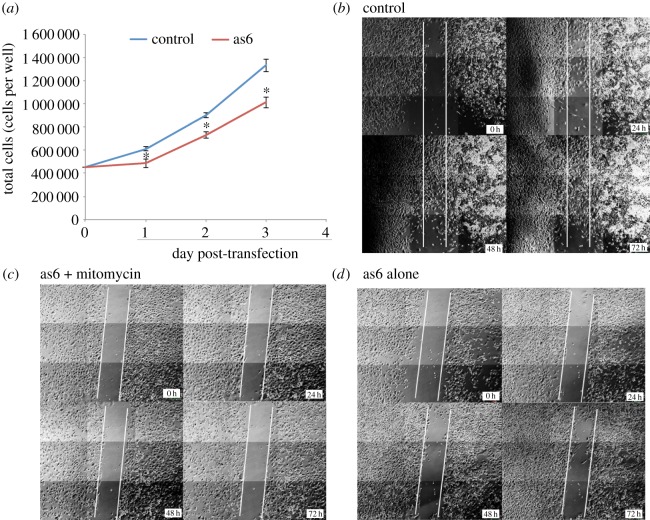
Phenotypic effect of Periostin TGS. (*a*) PC3 cell numbers following as6 treatment. Cells were counted every 24 h post-transfection with as6 or parent U6M2 plasmid. *p<0.05 by two-tailed *t*-test. Error bars indicate s.e.m. (*b*–*d*) Scratch assay 72 h after transfection with (*b*) parent U6M2 plasmid alone, (*c*) as6 expressing U6M2 treated with mitomycin C and (*d*) as6 expressing U6M2 plasmid alone. Cells were photographed at times 0, 24, 48 and 72 h post-transfection. The white lines indicate the initial scratch.

In an effort to determine if the sasRNA as6 functionally modulates the metastatic ability of tumour cells, we set out to investigate the observable phenotypic effects of as6 when introduced to tumour cells *in vitro*. During the generation of stable HEK293 and PC3 cell lines expressing the as6 sasRNA using Geneticin (G418) selection, it was discovered over a 6-week period that those cells that survived the selection process in both cell lines were unable to proliferate. As such, the generation of stable cell lines was not feasible, possibly due to the suppression of Periostin by as6. To explore this notion further cell counts in as6-treated versus -untreated PC3 cells were followed up over a 72 h period. Cultures treated with as6 demonstrated a significant reduction in exponential growth (figure 3*a*). Rather than a continued rate of exponential expansion, cell growth was observed to level off and undergo a more linear growth rate after only 3 days of as6 targeting. Notably, by the third day, there were 30% more cells in control samples than in those treated with as6, suggesting as6-directed suppression of Periostin impairs cell growth.

Periostin is known to be involved in cell metastasis [36,37]. To explore the ability of as6 to functionally modulate cell motility and metastasis, a scratch assay was performed on as6- versus control-treated PC3 cells (figure 3*b*–*d*). Examination of PC3 cell growth into a scratch over a 72-h period demonstrated severely inhibited growth of cells into the scratch in as6-treated cells relative to control cells (figure 3*b*,*d*). After 3 days, control cells were seen to move and proliferate into the scratch, covering over the gap left by the plastic insert (figure 3*b*). By contrast, the as6-treated cells showed very little movement into the provided space (figure 3*d*). The addition of mitomycin C to as6-treated cells caused the scratch to remain completely intact, with no edge distortion occurring, suggesting that the regrowth seen without mitomycin C in as6-treated cells was presumably due to cell migration rather than proliferation (figure 3*c*). These data suggest that as6 sasRNA-directed TGS of Periostin appears to be a potent suppressor of cell migration and is equivalent to the suppression of cell division by mitomycin C.

Recent studies have presented the concept of endogenous regulatory mechanisms directed by long non-coding RNAs (lncRNAs) in human cells which are responsible for altering the epigenetic state of a target locus, thus effecting the transcriptional expression of a protein coding gene [14,17,38]. This mechanism can be usurped when a sasRNA guide is introduced into the cell, bypassing endogenous lncRNA regulation of the target locus and inducing TGS (figure 1*a*) [13,19,32]. RNA-directed TGS has been observed in human cells before and functions by the sasRNA binding to target low-copy promoter-associated transcripts upstream or overlapping the sasRNA-targeted promoter [16,33], resulting ultimately in the recruitment of epigenetic remodelling complexes to the selected site of interest [13,16] and gene silencing. The advantage of sasRNA-directed TGS over other RNA-based silencing mechanisms, such as RNAi, is that TGS can be long-lasting and heritable. Only a short duration of sasRNA targeting to a gene promoter is required and once DNA methylation is recruited the targeted gene can stay repressed indefinitely [21]. The data presented here suggest that as6 sasRNA is capable of inducing TGS at the 5′UTR/promoter of Periostin and that this targeting has distinct phenotypic effects on cell motility. Collectively, the observations presented here suggest that as6 has the potential to function as an inhibitor of cell motility and possibly as an anti-metastatic molecule that could be highly useful in cancer treatments, although the effect of as6 on cell metastasis remains to be determined and will require further *in vivo* studies.

Previous observations demonstrated a correlation between high expression of Periostin and poor cancer-patient prognosis [8–12]. This is presumably due to Periostin’s ability to regulate tumour cell invasion and metastasis, as tumour metastasis is associated with the highest rate of mortality for cancer patients [36,37]. Suppression of Periostin gene expression *in vitro* via indirect methods [39–41], and others directly through RNAi [42–44], demonstrated a direct correlation between Periostin expression and the ability of cells to undergo migration and cell division. While previous studies using RNAi to suppress Periostin clearly demonstrated a loss of cell metastasis, the observed effects are only transient as the targeting of Periostin was at the post-transcriptional level [42–44]. The data presented here suggests that Periostin is susceptible to sasRNA-directed TGS and that it may be possible to stably suppress Periostin [13,17].

Secondary tumours, those that metastasize to new locations, are incapable of surviving without the presence of Periostin [45]. This matches with phenotypic data from the scratch assay and cell count, as well as the inability to generate stable cell lines, as as6 expressing cells would not proliferate and showed very limited ability to migrate, yet they did not undergo apoptosis. Periostin is also required to initiate alteration of the extracellular matrix, creating a niche environment that allows tumour invasion and proliferation [45,46]. The observed inability of the as6-treated cells to divide and migrate suggests the involvement of Periostin in rearranging the extracellular matrix and providing an environment conducive to metastasis. When suppression of this Periostin is induced by TGS, there is no alteration of the surrounding environment to favour cell invasion.

While the TGS induced by as6 was shown to be an effective inhibitor of metastatic and invasive potential of tumour cells, an effective method for introduction of the sasRNA into human patients and specifically to the tumours is required before it can be used to treat cancer patients. New delivery mechanisms that are targeted specifically to diseased cells are required if future treatment employing sasRNA-directed TGS is to become a viable option. However, once the sasRNAs reach their targets they have the distinct potential to provide stable epigenetic modifications to the gene promoter of interest and long-term heritable epigenetic silencing.

## Supplementary Material

Supplemental materials and methods

## Data accessibility

Data are available from the Dryad respository via http://dx.doi.org/10.5061/dryad.dc3m7.
