# Supplementary material for: RNA-directed epigenetic silencing of Periostin inhibits cell motility
Source: R Soc Open Sci. 2015 Jun 9;2(6):140545. doi: 10.1098/rsos.140545 (PMC4632543; doi:10.1098/rsos.140545)
Supplement: Supplemental materials and methods [file rsos140545supp1.docx]

**Methods and Supplemental Data for RNA-directed epigenetic silencing of Periostin inhibits cell metastasis**

**MATERIALS AND METHODS**

**Cell Culture**

PC3 cells were cultured at 37°C in 5% CO_2_ using DMEM supplemented with 1% penicillin/streptomycin and 10% FBS.

**sasRNA’s Expressing Constructs**

Small antisense RNA (sasRNA) sequences were generated and subsequently cloned into the U6M2 plasmid via *BGl*II and *Kpn*I restriction sites and validated using qRT-PCR as previously demonstrated [[1](#_ENREF_1)]. The sasRNA expressing constructs were transfected into PC3 cells using Lipofectamine 2000, using a ratio of 1µg/10^6 cells (Life Technologies, Carlsbad, CA, USA).

**RNA Extraction & cDNA Synthesis**

RNA extraction was performed using an RNeasy Mini Kit (Qiagen, Redwood City, CA, USA) and subsequently DNase treated (TURBO DNase, Life Technologies Carlsbad, CA, USA). Complimentary DNA (cDNA) was synthesised using 1µg cellular RNA in conjunction with M-MLV Reverse Transcriptase (Sigma/Aldrich, St Louis, MO, USA), dNTP’s (New England Biolabs, Ipswitch, USA) and random hexamer (Sigma/Aldrich, St Louis, MO, USA).

**qRT-PCR**

Quantitative RT-PCR (qRT-PCR) analysis was performed using KAPA SYBR FAST qPCR Master Mix (KAPA Biosystems, Woburn, MA, USA) and the desired primer set and cDNA. Plate was placed in ViiA 7 Real-Time PCR system (Life Technologies, Carlsbad, CA, USA). Cycling conditions were set up as follows: 95°C for 2 mins, then 40 cycles of 95°C for 3s and 60°C for 30s.

**Stable Cell Lines**

Stable cell lines were generated using the antibiotic Geneticin (800µg/ml)(Life Technologies, Carlsbad, CA, USA). PC3 and HEK293 cells were transfected with the desired plasmid, which had undergone linearization via *Sca*I enzyme digestion. The selection process was maintained for six weeks, and successful uptake of the plasmid resulted in the continued survival of the cell.

**Nuclear Run-On**

PC3 cells were transfected with either as6 expressing or parent U6M2 plasmid. Cell pellets were collected 72 hours later, followed by cell lysis (10 mM Tris- HCl, pH 7.4, 10 mM NaCl, 3 mM MgCl2, 0.5% NP-4) and subsequent resuspension (50 mM Tris-HCl, pH 8.3, 0.1 mM EDTA, 40% glycerol, 5 mM MgCl2). Samples were then flash frozen in liquid nitrogen. Upon thawing, in vitro transcription was performed using 50 μl glycerol suspension and 60 μl 2x transcription buffer (20 mM Tris-HCl, pH 8, 5 mM MgCl2, 300 mM KCl, 4 mM DTT, 2 mM ATP, 2 mM CTP, 2 mM GTP, 1 mM biotin-16-AA-5′-UTP, 100 U RNaseOUT (Invitrogen)). Samples were incubated for 45 mins at 30°C before being DNase treated. Nuclei were lysed (50 mM Tris-HCl, pH 7.4, 5% SDS, 0.125 M EDTA) and Proteinase K treated. RNA extraction was performed, and biotinylated RNA pulled down using Dynabeads M-280 Streptavidin (Life Technologies, Carlsbad, CA, USA). Beads were resuspsended in H_2_O, allowing cDNA synthesis directly off the beads and subsequent qRT-PCR analysis.

**Chromatin Immunoprecipitation**

As in nuclear run-on, cell pellets were collected 72 hours following transfection, resuspended in PBS, cross-linked with 1% formaldehyde and quenched with 0.125M glycine. Cell lysis (5 mM PIPES, pH 8, 85 mM KCl, 0.5 % NP40) was followed by nuclei lysis (50 mM TRIS-HCl, pH 8, 10 mM EDTA, 1% SDS) and sonication with a Q700 Sonicator (Qsonica). Samples were incubated at 4°C overnight with the desired antibody. Dynabeads Protein G (Life Technologies, Carlsbad, CA, USA) were used to pull down desired antibody, following wash steps in high salt twice (20 mM Tris-HCl, pH 8.1, 500 mM NaCl, 2 mM EDTA, 1% Triton X-100, 0.1% SDS), lithium chloride (10 mM Tris-HCl, pH 8.1, 0.25 M LiCl, 1 mM EDTA, 1% sodium deoxycholate, 1% NP40) and TE buffer twice (10 mM Tris-HCl, 1 mM EDTA). DNA/protein is eluted off the beads with 200μl ChIP elution buffer (100mM NaHCO_3_, pH 8, 1% SDS) and heated to 65°C for 10 minutes. Beads were removed from samples, which were then reverse cross-linked, RNase A and Proteinase K treated. Samples were cleaned up using PCR purification kit (Qiagen, Redwood City, CA, USA) and analysed by qRT-PCR. Antibodies used for ChIP (1 µg/sample) were for RNA Polymerase II (Abcam, Cambridge, MA, USA)(Abcam, cat. no. ab5095) and DNMT3a (Abcam, cat. no. 13888). Determination of relative enrichment is determined by contrasting amplification of a standard curve of genomic PC3 DNA. The bead alone no antibody control samples are subtracted from both the input and CHIPs and the relative enrichment determined as a fraction of input for the respective samples.

**DNMT3a and HDAC Inhibition**

Cells were transfected with desired plasmid, and 12 hours post-transfection both Trichostatin A (TSA) a verified HDAC inhibitor and 5-Azacytadine (5’ Aza-C) a verified DNA methyltransferase inhibitor were added to final concentrations 0.05μM and 4μM respectively [[2](#_ENREF_2)]. These compounds were then readministered every 24 hours. Seventy-two hours after the initial addition of TSA and 5’ Aza-C, RNA extraction was performed, followed by DNase treatment, reverse transcription and qRT-PCR.

**RNA Pol II Inhibition**

Twelve hours following transfection with desired plasmid, alpha-Amanitin drug was added to cells to final concentration 0.1 μg/ml [[3](#_ENREF_3)]. Seventy-two hours after drug addition, RNA extraction was performed, followed by DNase treatment, reverse transcription and qRT-PCR.

**Polyacrylamide Gel**

A 6% polyacrylamide gel was made up with 8 ml 1x Tris/Borate/EDTA (TBE) Buffer, 1.5 ml 40% acrylamide, 100 μl ammonium persulfate (APS) and 10 μl Tetramethylethylenediamine (TEMED). The gel was allowed to set for 20 minutes. qRT-PCR samples from control and as6 treated cells were loaded with gel loading dye onto gel, which was post-stained with ethidium bromide after completion of run and imaged using Geldoc Imaging System (BioRad).

**Cell Count**

Cell pellets were collected 72 hours following transfection and resuspended in 1ml PBS. A 10 μl aliquot was added to 10 μl 0.4% trypan blue. Viable cells were counted using a haemocytometer.

**Scratch Assay**

To a treated 24-well glass bottom plate, sterile micro-inserts were stuck to the bottom of each well. Plate was seeded with PC3 cells starved of FBS. After 24 hours, media was aspirated and 1ml of new DMEM media plus 10% FBS plus 1% penicillin-streptomycin was added. Cells were then transfected with desired plasmid. 12 hours following transfection, Mitomycin C was added to half of the wells from both control and treatment conditions to a final concentration of 20μg/ml. Sterile micro-inserts were then aseptically removed from wells. Photographs were taken of the cells every 15 minutes for 72 hours by a Nikon Eclipse TiE inverted microscope utilising phase contrast microscopy at the Biomedical Imaging Facility (BMIF), UNSW. This microscope contains a stage with a 37°C 5% CO_2_ chamber, allowing for maintenance of the cells during cell imaging.

**SUPPLEMENTAL DATA**

**Table S1** Sequences for sasRNA’s Targeted to Periostin Promoter

**sasRNA Sequence (5’ -- > 3’)**

as1 GATCTGATGCAGTGTTCCCTCCCACAATTTTTGGTAC

as3 GATCTGAATTTGAAGTTGCCGATGCTTCCTGGCTTTTTGGTAC

as4 GATCTGTTCAGACTCTCAGGTTGATGCTTTTTGGTAC

as5 GATCTGCAACTCTGACATGTATATAAATTCTTTTTGGTAC

as6 GATCTGTTCCCTCCCACAACTCTGACATGTTTTTGGTAC

**Figure S1** Promoter Sequence for Periostin Used to Generate sasRNA’s

AGCAGCAATAGTAGAGAAAACATGGGTAAAAAGGGAATCATCTTGAGTCTCTCCGTTGCAGTTAGTCCCCGAAGAGAACTGGCAGTGGGCTTTGGAGAGCTCAGAATTTATATACATGTCAGAGTTGTGGGAGGGAACACTGCATCAACCTGAGAGTCTGAACTCTTTCCAGGAAGCATCGGCAACTTCAAATTGCCAGCTAAGATTTGTTTTGGTTAGTTATATTTAAGGAACACATTGAGCTACTTTTCCTTTTCATTATAAAGAAGGTACTTATTTTTAATCATATGAACAATTGACTCACTGCATGTTTAAGTCTCAGTTTTATTCCTTTGTATCTTAGAGGGTTTTTAAG

**Table S2** Oligonucleotides Used In Current Study

**Periostin Primers**

>POSTN Forward

GAGCTTTACAACGGGCAAATAC

>POSTN Reverse

CTCCCTTGCTTACTCCCTTTC

**RPL10 Primers**

>RPL10 Forward

CCTCTTTCCCTTCGGTGTG

>RPL10 Reverse

AATCTTGGCATCAGGGACAC

**Periostin Promoter (for ChIP) Primers**

>POSTNpro Forward

AGTGGGCTTTGGAGAGCTCAGAAT

>POSTNpro Reverse

TTTGAAGTTGCCGATGCTTCCTGG

**REFERENCES**

1. Ackley, A., et al., *An Algorithm for Generating Small RNAs Capable of Epigenetically Modulating Transcriptional Gene Silencing and Activation in Human Cells.* Mol Ther Nucleic Acids, 2013. **2**: p. e104.

2. Morris, K.V., et al., *Small interfering RNA-induced transcriptional gene silencing in human cells.* Science, 2004. **305**(5688): p. 1289-92.

3. Weinberg, M.S., et al., *The antisense strand of small interfering RNAs directs histone methylation and transcriptional gene silencing in human cells.* RNA, 2006. **12**(2): p. 256-62.
